# Post-informal caregiver’s perspectives and experiences of the support network: a systematic literature review and meta-synthesis*

**DOI:** 10.1590/1980-220X-REEUSP-2024-0047en

**Published:** 2024-11-08

**Authors:** Catarina Inês Costa Afonso, Ana Cristina Spinola Madeira, Susana Pinto Leite Vasconcelos Teixeira de Magalhães

**Affiliations:** 1 Instituto Politécnico de Santarém, Escola Superior de Saúde, Centro de Investigação em Tecnologias e Serviços de Saúde, Porto, Portugal.; 2 Universidade do Porto, Instituto de Investigação e Inovação em Saúde (i3S), Porto, Portugal.

**Keywords:** Caregivers, Life Change Events, Community Networks, Systematic Review, Cuidadores, Acontecimientos que Cambian la Vida, Redes Comunitárias, Revisión Sistemática

## Abstract

**Objective:**

To examine and synthesize the evidence of experiences and perspectives on the specific context of informal post-care and the existing support network.

**Method:**

This is a qualitative systematic review with metasynthesis, according to the framework of the JBI, carried out in the CINAHL, LILACS, MEDLINE, BVS and PsycINFO databases, with no time limit. The articles were evaluated using the JBI Qualitative Data Extraction Tool. A total of 1,236 articles was identified, of which 18 were selected and 7 were analyzed. The level of evidence found was moderate.

**Results:**

The experiences and perspectives of the informal post-caregiver reveal the need for support, namely: in *personal development* - attribution of meaning, in self-care perspectives and in identity reconstruction; in *managing the impact of the legacy of caring* - discontinuity of support in the trajectory of caring, financial vulnerability, and the impact on mental health; *formal and informal resources for the future* - projection of the future, structured services and family and community support.

**Conclusion:**

Post-caregivers’ perception of their support network is that their individual and interpersonal needs are not identified, and that formal and informal services are not articulated.

## RESUMEN

**Objetivo:**

Examinar y sintetizar evidencia de experiencias y perspectivas sobre el contexto específico del poscuidador informal y la red de apoyo existente.

**Método:**

Se trata de una revisión sistemática cualitativa con metasíntesis, según el marco del JBI, realizada en las bases de datos CINAHL, LILACS, MEDLINE, VHL y PsycINFO, sin límite de tiempo. Los artículos fueron evaluados utilizando el Instrumento de Extracción de Datos Cualitativos del JBI. Se identificaron 1.236 artículos, de los cuales se seleccionaron 18 y se analizaron 7. El nivel de evidencia encontrado fue moderado.

**Resultados:**

Las experiencias y perspectivas de los post-cuidadores informales revelan la necesidad de apoyo en: *desarrollo personal* – atribución de significado, desde las perspectivas del autocuidado y la reconstrucción de la identidad; *gestión del impacto del legado de la atención* – discontinuidad del apoyo en la trayectoria de atención, vulnerabilidad financiera y el impacto en la salud mental; *recursos formales e informales para el futuro* – proyección de futuro, servicios estructurados y apoyo familiar y comunitario.

**Conclusión:**

La percepción de los post-cuidadores respecto de la red de apoyo es una falta de identificación de sus necesidades individuales e interpersonales, así como una desarticulación de los servicios formales e informales.

## INTRODUCTION

The demographic projection carried out by Eurostat, for the period between 2010 and 2060, should result in an increase of around 60% in the total dependency ratio. The growth of older people will be four times greater than that of the young population^(1)^. Associated with this data, the levels of dependency of older people are growing, and so is the need for informal caregivers^(1,2)^.

According to the National Alliance for Caregiving^(2)^, the informal caregiver is someone with a significant personal relationship with the person being cared for, such as a partner, a neighbor, or a friend, who performs an unpaid role, which arises when faced with the need to respond to difficulties in carrying out daily life activities^(3,4,5,6,7)^. Taking on the role of informal caregiver implies changes in the personal sphere as well as in professional life. This way, the informal caregiver leads a care trajectory characterized by transitions, simultaneously with the trajectory of dependence and/or illness of the person being cared for, with both touching each other and causing changes between them. These changes interfere with the informal caregiver, potentially making them isolated and separated from their support network. It is recognized that the readjustment or change persists until the cessation of care, when he/she becomes a post-informal caregiver^(3,4,5,6,7)^.

The literature highlights, in relation to the post-informal caregiver, difficulties related to: the grieving process, the resumption of social relationships, and financial changes. The low level of support for health is also noteworthy, as it can trigger problems or make them consumers of health services in a cycle of continuous dissatisfaction^(8,9,10,11,12)^. Regarding the social support network of the post-informal caregiver^(7)^, three dimensions are identified: *integration* (existence of social relations); *social media* (structure of social relations); and *support* (functional content of social relationships, including emotional, instrumental and informational), considered fundamental for the development of support networks. This way of looking underlies, in Watson’s theory of human care, an approach to the person as a whole, including what surrounds them^(13)^. Thus, the informal post-caregiver’s social support network includes everything that surrounds them and is part of their overall situation, that is, quality of life, health gains, contact with the community, with the family, reintegration into the job market, economic support, occupation, emotional well-being, connection with health services, and support for other caregivers (volunteering).

Recognizing the experience and perspectives of caring that marked the post-caregiver’s life and the changes in the social support network, it is now important to aggregate knowledge of these experiences in relation to the social support network and identify the constructs of its structure, as well as the specific context of the post-informal caregiver. The experience and perspectives of caring that marked life remain and the support network based on care disappears. Recognizing them can be an important strategy for highlighting gaps and providing guidance for action proposals. Knowledge of these experiences in relation to the social support network will allow us to identify the constructs of its structure, as well as the specific context of the post-informal caregiver. Through studies focusing on the post-caregiver’s experience, perspective and life events, we will seek to access the meaning of the social support network.

In this context, the objective is defined as: to examine and synthesize the evidence of experiences and perspectives on the informal post-caregiver support network, using the systematic review methodology with meta-synthesis, according to the JBI reference^(14,15)^.

## METHOD

### Design of Study

This is a systematic review with meta-synthesis of qualitative studies, conducted according to the JBI guidelines^(15)^, according to a protocol, a priori, registered on the PROSPERO platform with number CRD42023482019. According to JBI reference^(14,15)^, the steps of this review are described in detail in the following sections.

### Inclusion and Exclusion Criteria

In the definition of inclusion/exclusion criteria, the mnemonic PICo was considered, which means Population, Phenomenon of Interest, and Context.

Population – post-informal caregivers: informal caregivers over 18 years of age who have ceased/interrupted providing care due to the death and/or institutionalization of the person receiving care. The term bereaved, bereaved caregiver, or grieving caregiver was therefore included.

Phenomenon of Interest – experiences and/or life perspectives of the post-informal caregiver, following Watson’s theory of human caring^(13)^, in an approach to the person as a whole, including the support networks and/or that included quality of life, contact with the community, with the family, reintegration into the labor market, economic support, occupation, emotional well-being, connection with health services and/or support for other caregivers (volunteering).

Context - Services and/or structures, group and/or contexts in the community.

Types of study - Studies that focus on qualitative data, including but not limited to concepts such as phenomenology, grounded theory, ethnography, and action research. Qualitative descriptive studies that describe the experience, or the effects of the experience, will also be considered.

Thus, qualitative studies developed with informal post-caregivers were selected, without defining a time period, because no previous reviews on the topic under study were identified and because the aim was to perform an integrated view of all available evidence on the topic under study. Texts available electronically in English, Portuguese and Spanish were considered, with authors being fluent in these three languages, as they are the most widely used scientific languages in the world. Peer-reviewed articles are also added as an inclusion criterion, seeking to select only articles that have already been previously evaluated for their quality.

### Search Strategy

A preliminary search was conducted in PROSPERO, MEDLINE, Cochrane Database of Systematic Reviews, and JBI Evidence Synthesis, which did not identify current or ongoing studies in this area of study. According to this methodology, the protocol was developed^(16)^ and registration was carried out with PROSPERO.

The initial search took place in the MEDLINE (PubMed) and CINAHL (EBSCO) databases to find articles on the topic, during the month of March 2023. Then, a definitive strategy was formally proposed for each of the databases included, which was adjusted based on the lexicons and specificities of each one. Regarding the evaluation and analysis of results, they took place between May and September 2023, with completion in November 2023.

The combination of descriptors (MeSH) presented in English was used to conduct the search in the PubMed database, as well as in other databases, with small adaptations, according to their specificities: “[caregiver OR grief OR bereavement] AND [Life Change Events OR life experience OR experience] AND [social network analysis OR community networks OR network]”; five databases were used: Medical Literature Analysis and Retrieval System Online (MEDLINE–PubMed); Cumulative Index to Nursing & Allied Health Literature (CINAHL); Latin American and Caribbean Literature in Health Sciences (LILACS); Virtual Health Library (BVS); PsycINFO of the American Psychological Association. No results were obtained in the last three databases.

In accordance with JBI guidelines^(14,15)^, the same descriptors were inserted into five gray literature databases (Google Scholar, Index to Theses, Digital Dissertations, CAPES Dissertation and Thesis Database, Portuguese Open Access Scientific Repository - RCAAP), with no record of articles that answered the review question.

To ensure the fidelity of the blind analysis, the Rayyan® application (Qatar Computing Research Institute, Doha, Qatar) was used, which favors organization and precision in the selection of studies. After this stage, the articles were read in full to define the final sample. The references of the studies included in this stage were analyzed. Any disagreements that arose between the reviewers (CA and AS) were resolved through discussion, or with a third reviewer (SM).

### Article Selection and Quality Assessment

**Figure 1:**
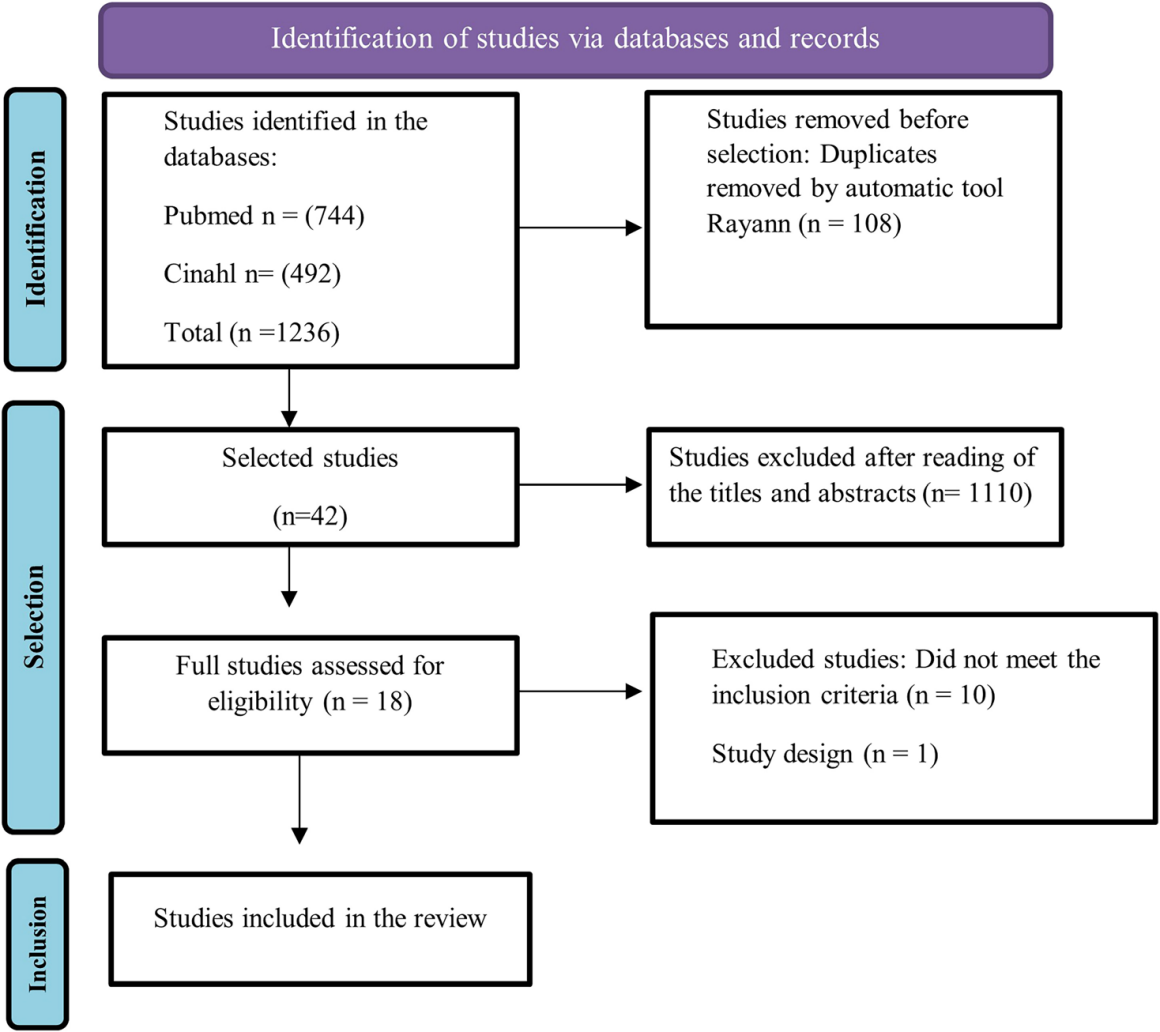
Flowchart for identification, selection and inclusion of meta-synthesis studies (PRISMA).

The selection process followed the Preferred Reporting Items for Systematic Reviews with Meta-synthesis (PRISMA) flowchart format^(17)^. The identified records were collected and transferred to Rayyan®. Resulting from the research strategy, 1,236 articles were found (744 in CINAHL, 492 in PUBMED). A total of 108 duplicate articles was removed. Subsequently, the titles and abstracts were read, and 1,110 articles were excluded. Eighteen articles were read in full, of which 10 were rejected because they did not meet the inclusion criteria and 1 because of the study design. The final sample consisted of 7 articles. The research results are presented in the form of a PRISMA flowchart^(17)^ (Figure 1). Databases where no results were obtained were not portrayed.

Data were extracted by two reviewers using the qualitative evidence tool for data extraction, containing details of the population, context, geographic location, study method, and phenomenon of interest. Data extraction took place in two ways, at the theme and subtheme level, contained in the selected articles and each discovery was extracted accompanied by an illustration, that is, a statement by the subjects who participated in the aforementioned research. The review team (CA and AS) analyzed each finding extracted and grouped them into categories, based on each one’s similarity of meaning. To facilitate visualization, it was initially described on sheets of paper and each reviewer (CA and AS) analyzed the similarities in meaning separately; then, a joint comparison work was carried out to reach consensus^(18)^. Disagreements were resolved by discussion and consensus with another reviewer (SM).

Eligible studies were critically appraised by two reviewers (CA and AS), who were independent in what regards methodological quality, using the JBI standard critical appraisal checklist^(15)^ for qualitative research, Critical Appraisal Checklist for Interpretive & Critical research (QARI). The team of researchers settled on a 70% cutoff point. The 7 articles have positive responses between 80% and 90% in QARI (Chart 1). The results of each of the studies included in their critical evaluation refer to questions related to the congruence between methodology, objectives, methods and evidence of ethical approval. We observed some limitations regarding the representation and analysis of results, the theoretical and cultural location of the researcher, and his/her influence on the research.

**Chart 1:** Critical evaluation of eligible studies – Santarém, Portugal, 2023.

Authors	Q1	Q2	Q3	Q4	Q5	Q6	Q7	Q8	Q9	Q1	QARI
Enyert e Burman, 1999^(19)^	Y	Y	Y	Y	Y	U	Y	Y	Y	Y	90%
Donovan et al, 2011^(20)^	Y	Y	Y	U	Y	Y	U	Y	Y	Y	80%
Pope, 2013^(21)^	Y	Y	Y	Y	U	Y	Y	Y	Y	Y	90%
Johansson et al, 2014^(22)^	Y	Y	Y	Y	Y	U	Y	Y	Y	Y	90%
MiIigan e Morbey, 2016^(23)^	Y	Y	Y	Y	U	Y	Y	Y	Y	Y	90%
Saunders e Groh, 2020^(24)^	Y	Y	Y	Y	U	Y	Y	Y	Y	Y	90%
Tay et al, 2021^(25)^	Y	Y	Y	U	Y	Y	U	Y	Y	Y	80%
	100%	100%	100%	80%	70%	80%	80%	100%	100%	100%	

Y = Yes, U = Unclear, N = No.

Q1. Is there congruence between the stated philosophical perspective and the research methodology?; Q2. Is there congruence between the research methodology and the research question or objectives?; Q3. Is there congruence between the research methodology and the methods used to collect the data?; Q4. Is there congruence between the research methodology and the representation and analysis of the data?; Q5. Is there congruence between the research methodology and the interpretation of the results?; Q6. Is there a statement locating the researcher culturally or theoretically?; Q7. Is the influence of the researcher on the research and vice versa addressed?; Q8. Are participants and their voices adequately represented?; Q9. Is the research ethical according to current criteria or, for recent studies, is there evidence of ethical approval by an appropriate body?; Q10. Are the conclusions drawn in the research report the result of the analysis or interpretation of the data?

### Data Analysis and Treatment

In data extraction, a level of credibility was established for each discovery, and the results were synthesized, representing the aggregation of data from primary studies^(18)^.

The results of the qualitative research were grouped according to the JBI meta-aggregation approach^(14,15)^. In this aggregation process, as established by the JBI^(14,15)^, the findings, due to descriptive and conceptual similarity, were aggregated into subthemes, which were later aggregated into broader and more comprehensive themes. In the search for similarities and differences between the different perspectives of the participants who experienced the phenomenon under study, the subthemes and themes that led to the finding of the meta-themes were integrated^(18)^. All findings were classified according to JBI credibility levels as “unequivocal” (I), “credible” (C) or “unsupported” (SS). Both reviewers (CA and AS) evaluated the extracted findings and reached agreement on their assigned levels of credibility. Findings accompanied by an illustration that is beyond reasonable doubt and therefore not open to challenge were assessed as “unequivocal.” Findings assessed as “credible” corresponded to those whose discoveries are accompanied by an illustration without a clear association and, therefore, open to challenge. “Unsupported” findings were those not supported by the data. Only findings classified as “unequivocal” or “credible” were included in the aggregation, as per the JBI approach^(15)^. From the classifications of findings and illustrations, the reviewers created categories that, when combined, produced a single comprehensive set of findings (Chart 2).

**Chart 2:** Primary data aggregation process – Santarém, Portugal, 2023.

Findings	Subthemes	Themes	Meta-theme
“No, it wasn’t difficult, like I said, I felt it was an honor to take care of her, to do what I could do.”^(19)^	Caring perspective (I)	Attribution of meaning (C)	**Support needs in personal development**
“I was too busy doing other things. And now, in real life, things matter more”^(19)^.	Vision of life (C)		
“Knowing that I took care of him the best way I could”^(24)^.	No resentment (C)		
“I meditate every night before I go to bed”^(24)^.	Time for me(C)	Self-Care Perspectives (C)	
“If you’re not exercising, you need to find something you enjoy doing physically. Find a way to relieve your stress, be creative and keep healthy and positive people around you”^(21)^.	Search for living conditions (I)		
“Keep my version of community and hopefully get into a situation where there will be a lot of people around, including people my age”^(21)^.			
“I often find that they don’t identify themselves as caregivers”^(23)^.	Exploring the identity of male caregivers (C)	Identity reconstruction (C)	
“The term counseling has a certain stigma and I would be wary of revealing the fact that I was receiving such a service to my male friends and coworkers”^(23)^.	Masculinity, emotion, and care (C)		
“I would be good if someone came in for a few more weeks to keep things back on track”^(24)^.	To keep being supported (I)	Discontinuity of support in the care pathway (I)	**Support needs in managing the legacy of care**
“Whether it’s a pastor or a nurse, just someone to talk to — that would have been helpful”^(24)^.			
“I feel isolated and the hospice cancelled the counselling session I had signed up for”^(25)^.	Loneliness and Grief in Isolation (I)		
“This (caregiving) has taken a huge toll on me financially. I’m living on less than half. I stopped buying magazines and other things we used to have. My biggest challenge is financial”^(24)^.	Weak financial resources(I)	financial vulnerability (I)	
“During the moments of respite, thoughts were still directed to my husband”^(24)^.	Persistence of concern in caring (C)	Impact on mental health (I)	
“Friends stopped coming here. I think that was the hardest part, not having family support. I walked away from everything because I couldn’t take care of him and maintain my social circles”^(24)^.	Lack of social relationships (I)		
“I’m alone, I miss him. Not having someone to care for or pamper is a negative aspect”^(24)^.	Sadness of Loneliness (I)		
“Then I was really down for a while”^(20)^.	Experience of loss (C)		
Unlike my parents, financially speaking, I hope that what we can do is move to a place where we can eventually support ourselves“^(21)^.	Plan for the future life (I)	Future projection (I)	**Needs of formal and informal responses for the future**
“I want to reconnect with people on an ongoing basis”^(24)^.	Building a network (I)		
“It was the support groups that helped me”^(24)^.			
“I have to tell you that dealing with bureaucracy is very, very frustrating and that is, you know, beyond the pain I’m feeling, it’s not fun. I wish someone had warned me of what was coming”^(25)^.	Moving forward in the face of uncertainty Managing the unknown (I)	Structured services (I)	
“The only time you get support is when you complain, after someone dies, and you should have it before that, to prepare for it”^(19)^.	Availability of support network (I)		
“I go to the support group every 15 days. It is useful for me to meet and share experiences with other people. We are, so to speak, at different stages of our caregiving situation, with our spouses in day care centers, in temporary care centers and in nursing homes”^(22)^.	Social support network (I)	Family and Community Support (I)	
“I sit here alone at the kitchen table and I feel extremely lonely. Visiting close friends is very important”^(22)^.			
“My faith prevents me from taking my own life”^(24)^.	Religious beliefs and survival strategies (C)		
The church is helping me, I’m doing well month to month, unless something comes up”^(20)^.	Spiritual Support (I)		
“If you understand the Dutch immigrants, they came with their families and basically all their time was spent in church, also their family and their friends. They don’t relate to anyone else”^(20)^.	Culturally informed care-seeking behaviors (I)		

(I) “unequivocal”, (C) “credible” or (SS) “unsupported”.

The process of grouping categories generates a meta-aggregation that is presented in a descriptive summary format, as shown in Chart 2. The results were used to support Chart 1, with a ConQual approach^(15)^, and the discussion stage, which includes recommendations according to GRADE^(15)^, the implications for practice, and the limitations of the review.

In the confidence assessment of the synthesized qualitative findings, the synthesized findings were classified according to the ConQual approach^(15)^. Each article is initially rated high, moderate, low, and very low – qualitative articles are rated high initially. From this starting point, they are evaluated based on reliability and credibility requirements^(15)^. Reliability is based on the first five questions of the critical appraisal instrument for qualitative studies. These questions are related to the adequacy of the research conduction, with its objectives and purposes. Depending on the number of “yes” answers to questions 1 to 5, the rating per article goes up or down (or remains the same): i) 4-5 “yes” answers, the rating remains unchanged; ii) 2-3 “yes” answers, the rating goes down by 1 level; iii) 0-1 “yes” answers, the assessment goes down 2 levels^(15)^. For credibility scoring, reviewers identified the level assigned to the findings that were synthesized by checking how many findings and what level was assigned. For each finding, reviewers assigned one of the following levels: Unequivocal (I); Credible (C); Unsupported (SS). The grading process considers the following score calculation for each synthesized finding: i) All unequivocal findings: grade remains unchanged; ii) Combination of unequivocal/credible findings: downgraded one level (-1); iii) Credible/unsupported findings: downgraded three (-3)^(15)^.

## RESULTS

The studies included in the sample were carried out between 1999 and 2021, one in Canada, one in Sweden, one in the United Kingdom, and four in the United States (Chart 3). The total sample of post-caregivers was 82 (Chart 3).

**Chart 3:** Summary table of the characteristics of the studies included in the meta-synthesis according to the authors, year of publication, country, participants, context, phenomenon of interest, and method of analysis - Santarém, Portugal, 2023.

Author/year	Country	Participants	Context	Phenomenon of interest	Analysis method
Enyert e Burman, 1999^(19)^	United States	7 informal post-caregivers; age 49-68;	Palliative care	Ability to transcend and find meaning in the experience of caring	Grounded Theory
Donovan et al, 2011^(20)^	Canada	7, post-informal caregivers (Reformed Christians); age 46-62;	Palliative Care	Experience of caring and mourning in a cultural context	Content analysis
Pope, 2013^(21)^	United States	15 post-caregiving women; age 50-65	Community, local agency for ageing support	Experience of caring for and planning own aging	Grounded Theory
Johansson et al, 2014^(22)^	Sweden	10 informal post-caregivers; age 56-86	Community, institutionalization of the person being cared for	Experience of caring in care transfer decisions	Content analysis
MiIigan e Morbey, 2016^(23)^	United Kingdom	15 male informal post-caregivers; age 56-86	Community Centers for Informal Caregivers	Experience of the impact of caring on the support network and identity	Thematic Analysis
Saunders e Groh, 2020^(24)^	United States	22 post-caregiver widows; average age 80 years	Community, group of widows from rural and urban settings	Experience of the impact of caring on the support network and widowhood	Thematic analysis
Tay et al, 2021^(25)^	United States	6 informal post-caregivers; average age 32-67;	Community, post COVID19	Experiencing the impact of caring in a time of social distancing	Content analysis

In accordance with the established objective, the systematic review allowed the subjective description of the experiences and perspectives of the post-informal caregiver regarding the support network after care. The review included 24 years of scientific production, identified and analyzed 7 qualitative studies. Nine (9) qualitative findings were extracted, of which six (6) were assessed as unequivocal and three (3) as credible.

**Figure 2:**
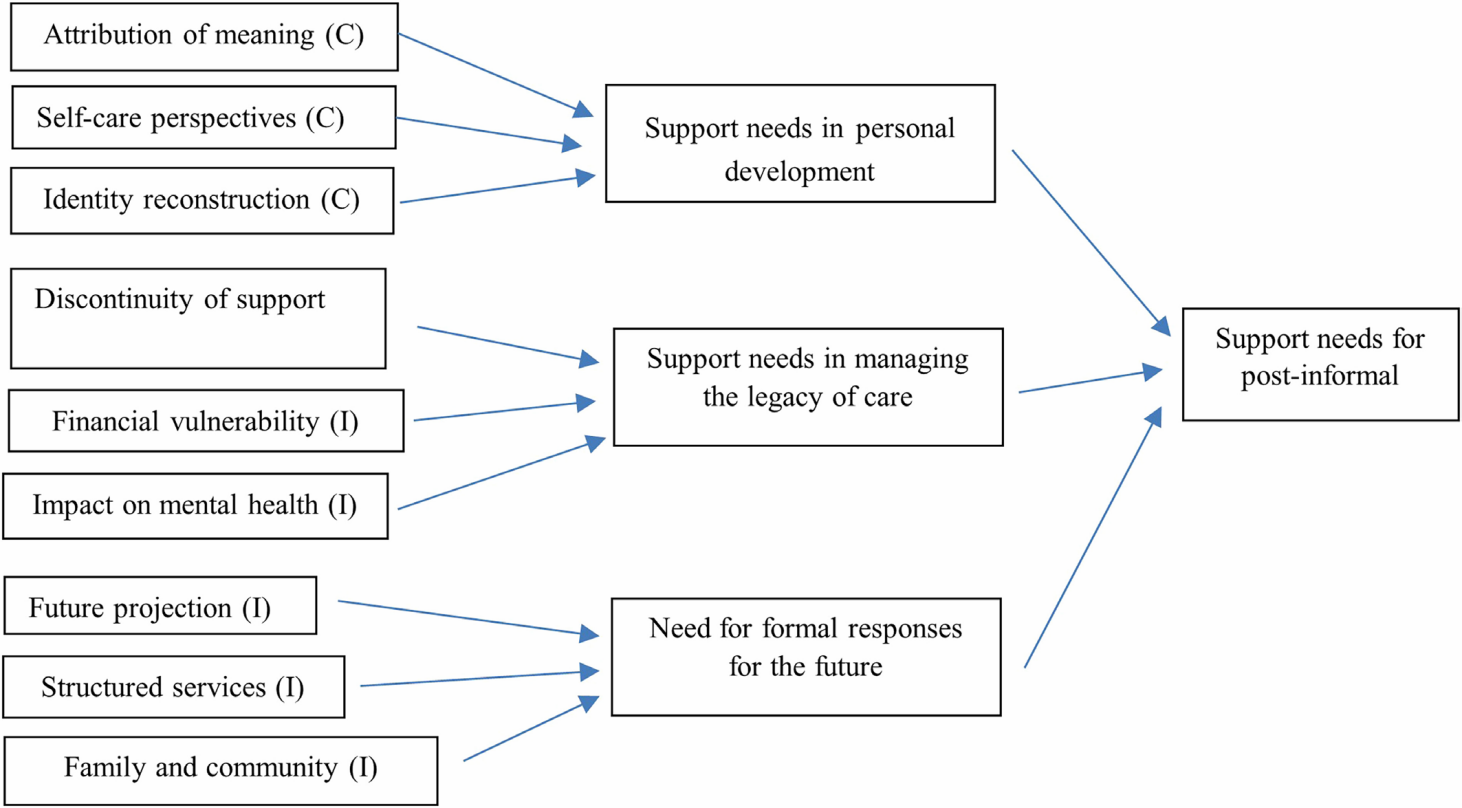
Meta-aggregation and meta-synthesis result in which I – Unequivocal and C – Credible.

The set of findings was grouped into one category and three subcategories constructed based on divergences, similarities, or complementarities. Figure 2 presents the grouping of categories that generated the following summary: the need for support from the informal post-caregiver.

Chart 4 shows the summary of the findings with the ConQual Score. The summary of findings presents high reliability, since the result of the studies quality evaluation presented a yes answer for more than 4 criteria. This rating provided a “moderate” degree of confidence for the synthesized finding, due to reliability issues (most studies had no statement of researcher location and no acknowledgement of author influence on the research). However, credibility was assessed as moderate, since the illustrations were classified as unequivocal and credible, that is, not all of them significantly represented the finding presented in the primary studies.

**Chart 4:** ConQual summary of findings – Santarém, Portugal, 2023.

Title: Experiences and perspectives of the post-informal caregiver in the support network				
Participants: informal post-caregivers Phenomenon of interest: experiences and perspectives of the support network Context: structures, group and/or contexts in the community				
**Summary of findings**	**Research type**	**Reliability**	**Credibility**	**ConQual Score**
Need for support for post-informal caregivers	Qualitative	High (4–5 “yes” answers)	Moderate (the combination of unequivocal/credible findings downgraded one grade (–1))	Moderate 6 (U) + 3 (C)

**Figure 3:**
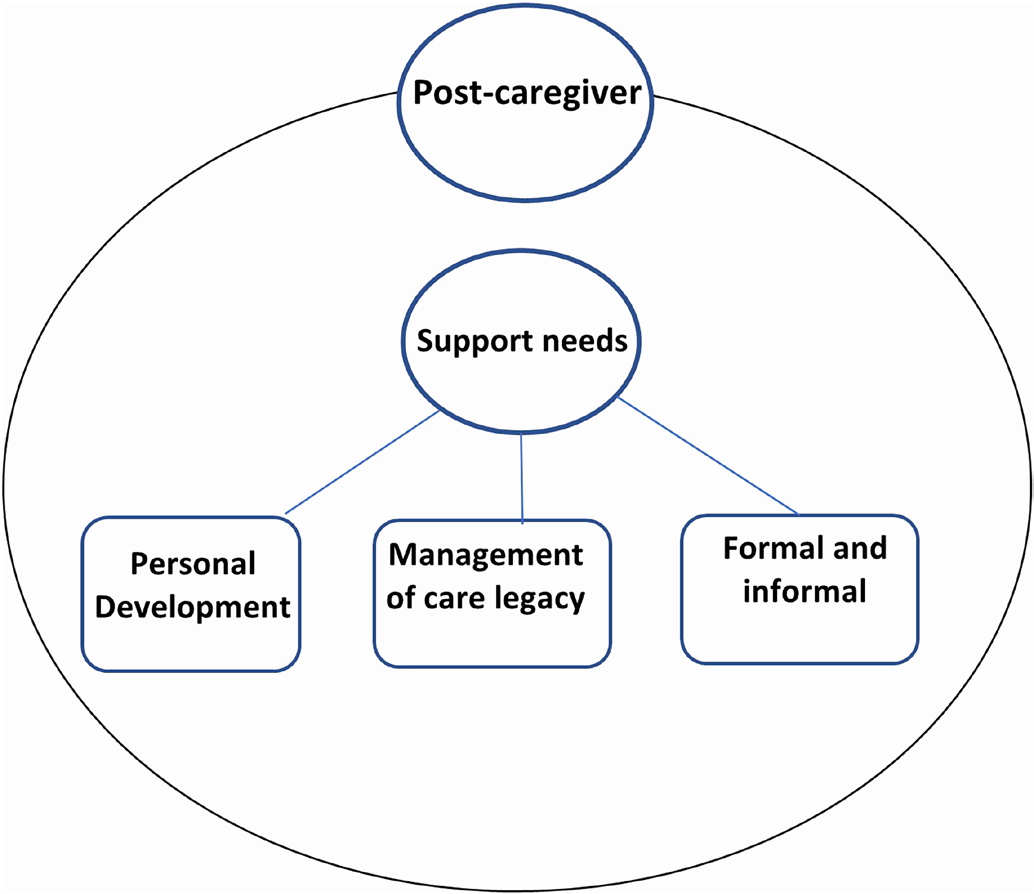
List of identified themes.

The analysis of the studies resulted in the category of need for post-caregiver support with three subcategories that supported the construction of the meta-aggregation, which will be presented below: 1) Need for support in personal development; 2) Need for support in managing the legacy of caring; 3) Need for formal and informal resources for the future (Figure 3).

### Need for Support in Personal Development

This theme brings together perceptions of the need for support in personal development. According to the studies included, the needs translate into: attribution of meaning, perspectives of self-care, and identity reconstruction.

The attribution of meaning for informal post-caregivers is characterized by the need for support in personal development from the perspective of caring, vision of life, and without/absence of resentment^(19,24)^.

The need to attribute meaning encompasses the search for meaning in what was experienced. In the different studies^(19,24)^ there is an idea that there was a pause in life to become a caregiver, and then, when one stops caring, the need to seek meaning for what was experienced becomes essential. The attribution of meaning in the vision of life was reinforced or reconfirmed by the experience of caring. The vision of life was used as a strategy to maintain or restore balance, resulting in a sense of coherence, during and after the care experience, which is intended to be without resentment^(19,24)^. This process puts into perspective what was experienced, recognizing its meaning, which favors the absence of resentment.

In the need for support for personal development, the perspective of self-care is also present, which is characterized by: time for myself (oneself) and the search for living conditions^(21,24)^.

For the post-caregiver, time for me is reverted to focusing on themselves, dedicating time to themselves, to carrying out activities aimed at their own well-being, such as meditation^(21,24)^. The search for living conditions is associated with self-care perspectives, as it expresses the movement that the post-caregiver maintains with other people/other activities^(21,24)^.

Identity reconstruction for informal post-caregivers is characterized by the need for support in personal development and is expressed in the identity of caregivers, masculinity, emotion, and care^(23)^. The analysis carried out allowed us to identify the identity of caregivers as essential for identity reconstruction and, consequently, for their personal development^(23)^. Adding masculinity vs femininity, emotion and care, aspects included in identity reconstruction^(23)^.

### Need for Support in Managing the Legacy of Caring

The need for support in managing the legacy of caring refers to marks, memories and changes associated with the role of informal caregiver that persist. This meta-theme brings together perceptions of the need for support in managing the legacy of care, which translate into: discontinuity of support in the care trajectory, financial vulnerability, and impact on mental health.

The discontinuity of support in the trajectory from caregivers to post-caregivers remains even after the loss. The importance for them of remaining being supported is expressed in the written discourse in the studies under analysis, particularly in women and their transition to widowhood^(24)^.

Loneliness from the perspective of isolation in mourning is associated with the absence of care, which are highlighted as factors that hinder the reestablishment of the meaning of life: lack of intimacy, adaptation to being alone, or ceasing to be a caregiver^(24)^.

Financial vulnerability and/or weak financial resources stand out as one of the greatest challenges pointed out by post-caregivers, influencing their life choices; however, in most situations, there was help in the different communities through support groups/networks^(24)^.

The impact on mental health resulting from the experience of informal caregiver is relevant to the meaning of life for the post-caregiver, with the transition occurring according to the life history of each person/family/community. The studies analyzed show that the feeling of concern for caring is present even after loss, the absence of social relationships, sadness and loneliness, feelings that remain for some time. The impact on the mental health of post-caregivers appears to influence the transition from caregiving to widowhood in two major themes: “time for me now” and “Loneliness”^(24)^.

### Formal and Informal Response Needs for the Future

This theme brings together perceptions of the need for formal and informal responses for the future. According to the studies included, the needs translate into: future projection, structured services, and family and community support. The projection of the future for informal post-caregivers is implicit in the need for formal and informal responses for the future and is characterized by the need to create a life project/plan and build a support network^(19,21)^.

The projection of the future encompasses the vision of life that follows in the social, community and financial plans^(19,21)^. In studies^(19,21)^, the idea that is present is that the role of informal caregiver makes the social situation vulnerable, such as the loss of close relationships – community relationships manifested by isolation from community relationships and activities – the financial situation, due to the loss of capital, low resources and difficulty in re-entering the job market. Building a network appears as a strategy for projecting the future, with the desire to resume interpersonal relationships, breaking with isolation and valuing participation in support groups^(19,21)^.

The absence of structured services demonstrates the need for support for formal and informal responses for the future, for managing the unknown, and for the availability of the support network^(19,25)^. Managing the unknown represents the lack of knowledge of what may happen after the death of the person receiving care. This need is related to the availability of the existing support network which, according to the studies analyzed, is not aligned with the demands of the post-caregiver^(25)^. Family and community support for informal post-caregivers includes social support network, religious beliefs, spiritual support, and family cultures^(20,22,24)^. In the studies analyzed, the social support network is essential in resuming interpersonal relationships and sharing experiences. Religious beliefs are identified as an anchor in the management of suffering and as a bridge to the relationship with the religious structure. Finally, family cultures prove to be fundamental in the feeling of safety and support, throughout the care journey^(20,22,24)^.

## DISCUSSION

This systematic review with meta-synthesis was conducted to better understand the experiences and perspectives of informal post-caregivers regarding the support network after caregiving. Seven (7) studies were included after a rigorous search and selection process. All studies included were of high quality based on the JBI critical appraisal checklist for qualitative research (Chart 1).

The scientific production included in the review took place between 1999 and 2021, and the studies included involve researchers from the USA, Canada, Sweden, and the United Kingdom (Chart 3). The quality of evidence is moderate (Chart 4).

It should be noted that no studies were carried out in Portugal, which is relevant, as the location of the studies may emphasize the concern with informal post-caregivers. Thus, it was possible to find that the interest in this theme involves researchers from countries on several continents, with different views on the phenomenon, considering cultural, economic, and social patterns in its understanding. Conversely, the reality of the countries under analysis highlights that the topic under study is still a high concern on countries where interest in the worsening of aging and informal care is valued. The context of the studies is community-based, in palliative care, and in an institutionalized context, highlighting the importance of family, friends and community structures such as the church^(19,20,21,22,23,24,25)^.

The meta-synthesis identified and provided an understanding of informal post-caregiver experiences and perspectives on the post-care support network and compared these with the literature available. The perception of post-caregivers regarding the support network is that their individual and interpersonal needs are not identified, as well as that formal and informal services are not articulated^(19,20,21,22,23,24,25)^.

The studies included in the review draw the attention to the need for post-caregivers to be supported in their personal development. These studies explore the experience of caring and how it changes the way life is perceived, highlighting the importance of valuing support in the reconstruction of identity and in the integration of the experience of caring into life after care. This finding corroborates the results of the study by Lennaerts-Kats et al.^(26)^, which aimed to understand the experiences of bereaved family caregivers during the period of informal care in the palliative care phase, as well as after the death of their loved one. In the same study, it was found that post-caregivers feel changes in their mental health for many years before and after the death of the person being cared for. The post-informal caregiver’s vulnerability, identified in the review, is in line with what is shown in previous studies^(3,4,5,6,7)^. Informal post-caregivers are identified as bereaved and/or former caregivers, with a prevalence of females^(19,20,21,23,24)^, although one study focused on male post-caregivers^(25)^. It was detected that the process of providing care and its impact on life after the cessation of care is manifest in the personal sphere, with disruptions in life and relationships. It was recognized that this process enhances the change in their social and relational roles, which can lead to distancing from their identity, crystallized in the role of caregiver, as well as changes in personal health^(19,24)^. In the interpersonal sphere, the loss of social relationships and the desire to resume social activity are clear, although the current reality is different from the reality when the role of caregiver is assumed^(19,20,21,22,23,24,25)^. This analysis highlights the importance of attending to and valuing the experience during the caregiver’s journey.

Regarding the findings on the need for support due to the impact of the legacy of caring, especially on the caregiver’s health, informal post-caregivers are referred to as having higher rates of stress^(27)^, depression^(28)^, and sleep problems^(29–30)^. In the same way, informal care disturbs sleep in various ways^(30,31)^, with sleep disturbances persisting after care is stopped. This fragility of the caregiver’s physical and mental health remains in the post-caregiver period with implications for the grieving process^(8)^. These aspects reinforce the importance of maintaining contact with healthcare professionals after the cessation of care.

The need for support in formal and informal resources is identified in the studies included in the review, through quality of life, health gains, contact with the community and family, reintegration into the labor market, economic support, occupation, emotional well-being, connection with health services, and support for other caregivers. The articles address the person as a whole^(7)^, highlighting the post-caregiver’s path of caring^(19,20,21,22,23,24,25)^. This need is identified in the community, highlighting the importance of family, friends, and community structures such as the church^(19,20,21,22,23,24,25)^. These references reinforce the framework of support for post-family caregivers in a community context.

During the systematic review with meta-synthesis, the relevance of evidence from qualitative studies on the experiences and perspectives of informal post-caregivers in the support network was verified, namely: need for support in personal development; need for support in managing the impact of the legacy of care; need for formal and informal resources. These findings confirm the necessity to embrace and qualify these experiences when structuring a support network. The meta-synthesis revealed gaps related to the need for support, such as in personal development, in managing the impact of the legacy of caring, and in formal and informal resources. Thus, with this study, the strategic proposal for developing the support network must settle on identified support needs.

Regarding implications for practice, the findings suggest that interventions focused on improving the support needs of post-caregivers may be beneficial in structuring a support network. The network pillars must be based on the support needs for personal development, on the management of the impact of the legacy of care, and on formal and informal resources for the future. The role of health professionals, in particular specialist nurses, is essential to encourage health surveillance, focusing on reflection and discussions about the experience, the perceived meaning, and also identifying potential support systems and availability of post-caregivers. This surveillance could be envisaged in a structured consultation by professionals trained in the specificities of the post-informal caregiver. The importance of integrating the support network from a holistic perspective^(13)^ of the post-caregiver should also be mentioned, looking for continuous responses in the community^(3,7)^.

However, recommendations are made with caution due to the moderate quality of the evidence (Chart 4). According to the results obtained, following the JBI GRADE^(14)^, it is recommended that (1) the support network for post-informal caregivers be in a community context^(19,20,21,22,23,24,25)^; (2) the needs for support in personal development, in managing the impact of the legacy of care and in formal and informal resources are met^(19,20,21,22,23,24,25)^; (3) the perceptions and experiences as caregivers are valued^(19,20,21,22,23,24,25)^.

Some limitations of the study are identified: 1) Reduced number of studies included. The team of investigators did not set a time limit, which shows the limited evidence in the area. There is also the possibility of missing some relevant studies in different languages. 2) The results are not intended to be generalizations and should be interpreted according to the structures, group and/or contexts in the community. The systematic literature review with meta-synthesis allowed, through various qualitative studies, the construction of increasingly robust knowledge with greater transferability in the area of informal post-caregiver. However, qualitative research provides theoretical and contextual insights into the experiences of a limited number of participants in community settings. 3) Possibility of polarization of results, as the experience of the support network has discrepant representations according to the experiences of post-caregivers and community contexts. However, the perceptions found in the studies allowed expanding the understanding of the phenomenon under investigation, as the results indicated that, in these contexts, the diversity of representations illustrates the need to include the support network in the holistic perspective of the post-caregiver. 4) The limitations described intensify the existing difficulties regarding the phenomenon and the need to carry out new studies in different contexts. The majority of studies included in the review did not address the influence of the environment and the researcher on the development of the research, an aspect that was not mentioned.

## CONCLUSION

This systematic review with meta-synthesis found that evidence from qualitative studies on the experiences and perceptions of informal post-caregivers of their support network reveals a lack of identification of their individual and interpersonal needs, as well as a lack of articulation between formal and informal services. The results are consistent with previous studies targeting informal caregivers that highlight the importance of social support. The findings of this meta-synthesis review emphasize the need to focus on the importance of support for post-caregivers, which confirms the value of embracing and paying attention to the needs of post-caregivers, such as support in personal development, support in managing the impact of the legacy of caring, and formal and informal resources.

Further studies are required to explore the experience of informal post-caregivers in different cultural contexts. Longitudinal studies could provide a deeper understanding of changes in experiences over time.

It is imperative that health professionals begin to discuss the specific needs of post-caregivers and structured responses, particularly the implementation of health surveillance consultations available in the national health service. This requires trained professionals, such as specialist nurses who can enhance the specific monitoring skills of informal post-caregivers. It is essential, at the level of nursing training, to introduce the theme of post-caregivers and their support network in the community into the curricula. Finally, it is suggested that future research be developed, aiming at finding the elements to be mobilized in the construction of the support network; at identifying the perceptions of professionals regarding the way the network is operationalized; and at implementing interventions that allow support and monitoring of the informal post-caregiver.

It is believed that the results of the meta-synthesis review will have an impact on the development of a culture of care rooted in the community, in which caring experiences are valued.

According to the results found, it seems desirable to develop research to a) identify strategic axes for the development of the support network, replicable and adaptable to community contexts; b) implement structured interventions based on the needs that allow the continuity of the post-caregiver monitoring by the health team.
