# Supplementary material for: Post-informal caregiver’s perspectives and experiences of the support network: a systematic literature review and meta-synthesis
Source: Rev Esc Enferm USP. 2024 Nov 8;58:e20240047. doi: 10.1590/1980-220X-REEUSP-2024-0047en (PMC11548915; doi:10.1590/1980-220X-REEUSP-2024-0047en)
Supplement: Translation

# Translation: Perspectivas e experiências do pós-cuidador informal da rede de suporte: revisão sistemática da literatura metassíntese*

**DOI:** 10.1590/1980-220X-REEUSP-2024-0047pt

**Authors:** Catarina Inês Costa Afonso, Ana Cristina Spinola Madeira, Susana Pinto Leite Vasconcelos Teixeira de Magalhães

**Affiliations:** 1 Instituto Politécnico de Santarém, Escola Superior de Saúde, Centro de Investigação em Tecnologias e Serviços de Saúde, Porto, Portugal.; 2 Universidade do Porto, Instituto de Investigação e Inovação em Saúde (i3S), Porto, Portugal.

**Keywords:** Cuidadores, Acontecimentos que Mudam a Vida, Redes Comunitárias, Revisão Sistemática

## Abstract

**Objetivo:**

Examinar e sintetizar a evidência das experiências e perspectivas sobre o contexto específico do pós-cuidador informal e a rede de suporte existente.

**Método:**

Trata-se de uma revisão sistemática qualitativa com metassíntese, conforme referencial do JBI, realizada nas bases de dados CINAHL, LILACS, MEDLINE, BVS e PsycINFO, sem limite temporal. Os artigos foram avaliados com uso do Instrumento de Extração de Dados Qualitativos do JBI. Identificaram-se 1.236 artigos, dos quais 18 foram selecionados e 7 foram analisados. O nível de evidência encontrado foi moderado.

**Resultados:**

As experiências e perspectivas do pós-cuidador informal revelam a necessidade de apoio em: *desenvolvimento pessoal* – atribuição de sentido, nas perspectivas de autocuidado e na reconstrução identitária; *gestão do impacto do legado de cuidar* – descontinuidade do apoio na trajetória do cuidar, a vulnerabilidade financeira e o impacto na saúde mental; *recursos formais e informais para o futuro* – projeção de futuro, serviços estruturados e apoio familiar e comunitário.

**Conclusão:**

A percepção dos pós-cuidadores relativamente à rede de suporte é de ausência de identificação das suas necessidades individuais e interpessoais, bem como de desarticulação dos serviços formais e informais.

## INTRODUÇÃO

A projeção demográfica realizada pelo Eurostat, para o período compreendido entre 2010 e 2060, deverá traduzir-se num aumento de cerca de 60% da razão de dependência total. O crescimento das pessoas idosas será quatro vezes superior ao da população jovem^(1)^. Associado a estes dados, crescem os níveis de dependência das pessoas idosas, e simultaneamente a necessidade dos cuidadores informais^(1,2)^.

Segundo a National Alliance for Caregiving^(2)^, o cuidador informal é alguém com uma relação pessoal significativa com a pessoa cuidada, como um companheiro, um vizinho ou um amigo, que desempenha um papel não remunerado, que surge perante a necessidade de dar resposta à dificuldade na realização das atividades de vida quotidiana^(3,4,5,6,7)^. A assunção do papel de cuidador informal implica alteração na esfera pessoal bem como na vida profissional. Deste modo, o cuidador informal protagoniza uma trajetória de cuidar caracterizada por transições, em simultaneidade com a trajetória de dependência e/ou doença da pessoa cuidada, sendo que ambas se tocam e provocam mudanças entre si. Estas mudanças interferem no cuidador informal, podendo torná-lo isolado e desagregado da rede de suporte. Reconhece-se que o reajuste ou mudança persiste até a cessação de cuidados, quando se torna pós-cuidador informal^(3,4,5,6,7)^.

A literatura evidencia, em relação ao pós-cuidador informal, dificuldades relacionadas com: o processo de luto, o retomar de relações sociais e as mudanças financeiras. Destaca-se igualmente a diminuta aposta no apoio à saúde, podendo desencadear problemas, ou torná-los consumidores dos serviços de saúde num ciclo de insatisfação contínua^(8,9,10,11,12)^. Em relação à rede de suporte social do pós-cuidador informal^(7)^, identificam-se três dimensões: *integração* (existência de relações sociais); *redes sociais* (estrutura das relações sociais); e *suporte* (conteúdo funcional das relações sociais, incluindo emocional, instrumental e informacional), consideradas fundamentais para o desenvolvimento de redes de apoio. Este modo de olhar está subjacente, na teoria do cuidado humano de Watson, numa abordagem à pessoa na sua globalidade, incluindo o que a envolve^(13)^. Desta forma, a rede de suporte social do pós-cuidador informal inclui tudo o que o rodeia e faz parte da sua globalidade, isto é, qualidade de vida, ganhos em saúde, contato com a comunidade, com a família, reintegração no mercado de trabalho, suporte econômico, ocupação, bem-estar emocional, ligação com os serviços de saúde e apoio a outros cuidadores (voluntariado).

Reconhecendo a experiência e as perspectivas de cuidar que marcaram a vida do pós-cuidador e as alterações da rede de suporte social, importa agora agregar o conhecimento destas experiências em relação à rede de suporte social e identificar os construtos da sua estrutura, bem como o contexto específico do pós-cuidador informal. A experiência e as perspectivas de cuidar que marcaram a vida permanecem e a rede de suporte assente no cuidado desaparece. Reconhecê-las pode ser uma importante estratégia para evidenciar as lacunas e orientar para propostas de atuação. O conhecimento destas experiências em relação à rede de suporte social permitirá identificar os construtos da sua estrutura, bem como do contexto específico do pós-cuidador informal. Por meio de estudos com foco na experiência, na perspectiva e nos eventos de vida do pós-cuidador, procurar-se-á aceder ao significado da rede de suporte social.

Neste enquadramento, define-se como objetivo: examinar e sintetizar a evidência das experiências e perspectivas sobre a rede de suporte do pós-cuidador informal, recorrendo à metodologia da revisão sistemática com metassíntese, conforme referencial do JBI^(14,15)^.

## MÉTODO

### Tipo de Estudo

Trata-se de uma revisão sistemática com metassíntese de estudos qualitativos, conduzida segundo as diretrizes do JBI^(15)^, de acordo com um protocolo, a priori, registrado na plataforma PROSPERO número CRD42023482019. Conforme referencial do JBI^(14,15)^, as etapas desta revisão estão descritas pormenorizadamente nas seções seguintes.

### Critérios de Inclusão e Exclusão

Na definição dos critérios de inclusão/exclusão, considerou-se o mnemônico PICo que significa a População, o Fenômeno de Interesse e o Contexto.

População – pós-cuidadores informais: cuidadores informais com mais de 18 anos que tenham cessado/interrompido o cuidado, por falecimento e/ou institucionalização da pessoa alvo de cuidados. Incluiu-se assim, o termo enlutado, cuidador enlutado ou em luto.

Fenômeno de Interesse- experiências e/ou perspectivas de vida do pós-cuidador informal, seguindo a teoria do cuidado humano de Watson^(13)^, numa abordagem à pessoa na sua globalidade, incluindo-se as redes de suporte e/ou que incluíam qualidade de vida, contato com a comunidade, com a família, reintegração no mercado de trabalho, suporte econômico, ocupação, bem-estar emocional, ligação com os serviços de saúde e/ou apoio a outros cuidadores (voluntariado).

Contexto – Serviços e/ou estruturas, grupo e/ou contextos na comunidade.

Tipos de estudo - Estudos que se centram em dados qualitativos, incluindo, mas não se limitando a concepções como a fenomenologia, a teoria fundamentada, a etnografia e a investigação-ação. Serão igualmente considerados os estudos qualitativos descritivos que descrevem a experiência, ou os efeitos da experiência.

Desta forma, foram selecionados estudos de abordagem qualitativa desenvolvidos com pós-cuidadores informais, sem definição de período temporal, porque não foram identificadas revisões prévias sobre a temática em estudo e por se pretender uma visão integrada de toda a evidência disponível sobre o tema em estudo. Consideraram-se textos disponíveis eletronicamente, nos idiomas inglês, português e espanhol, sendo os autores fluentes nessas três línguas, e por elas configurarem-se como as línguas científicas mais utilizadas no mundo. Acrescenta-se igualmente, como critério de inclusão, artigos com peer review, procurando-se selecionar apenas artigos já previamente avaliados quanto à sua qualidade.

### Estratégia de Busca

Desenvolveu-se uma pesquisa preliminar, na PROSPERO, MEDLINE, Cochrane Database of Systematic Reviews, e JBI Evidence Synthesis, que não identificou estudos atuais ou em desenvolvimento nesta área de estudo. De acordo com esta metodologia, desenvolveu-se o protocolo^(16)^ e realizou-se o registro na PROSPERO.

A pesquisa inicial ocorreu nas bases de dados MEDLINE (PubMed) e CINAHL (EBSCO) para identificar artigos sobre a temática, no decorrer do mês de março 2023. Em seguida, foi formalmente proposta uma estratégia definitiva para cada uma das bases de dados incluídas, sendo esta ajustada tendo por base os léxicos e especificidades de cada uma. Quanto à avaliação e análise dos resultados, ocorreram entre maio e setembro de 2023, com complementação em novembro de 2023.

A combinação de descritores (MeSH) que se apresenta em inglês foi usada para conduzir a busca na base de dados PubMed, assim como nas demais bases de dados, com pequenas adaptações, de acordo com as suas especificidades: “[caregiver OR grief OR bereavement] AND [Life Change Events OR life experience OR experience] AND [social network analysis OR community networks OR network]”; Foram utilizadas 5 bases de dados: Medical Literature Analysis and Retrieval System Online (MEDLINE–PubMed); Cumulative Index to Nursing & Allied Health Literature (CINAHL); Literatura Latino-Americana e do Caribe em Ciências da Saúde (LILACS); Biblioteca Virtual em Saúde (BVS); PsycINFO da American Psychological Association. Nas três últimas bases de dados não se obtiveram resultados.

Em conformidade com as orientações do JBI^(14,15)^, os mesmos descritores foram inseridos em cinco bases de dados de literatura cinzenta (Google Scholar, Index to Theses, Digital Dissertations, Banco de dissertações e teses da CAPES, Repositório Científico de Acesso Aberto de Portugal - RCAAP), sem registro de artigos que respondessem à questão de revisão.

Para garantir a fidelidade da análise às cegas, foi utilizado o aplicativo Rayyan® (Qatar Computing Research Institute, Doha, Qatar), que favorece a organização e precisão na seleção dos estudos. Após essa etapa, realizou-se a leitura dos artigos na íntegra, para a definição da amostra final. Foram analisadas as referências dos estudos incluídos nessa etapa. Quaisquer divergências que surgiram entre os revisores (CA e AS) foram resolvidas por meio de discussão, ou com um terceiro revisor (SM).

### Seleção Dos Artigos e Avaliação da Qualidade

O processo de seleção seguiu o formato do fluxograma Preferred Reporting Items para revisões sistemáticas com metassíntese (PRISMA)^(17)^. Os registros identificados foram recolhidos e transferidos para o Rayyan®. Resultante da estratégia de pesquisa, foram encontrados 1.236 artigos (744 na CINAHL, 492 na PUBMED). Foram removidos 108 artigos duplicados. Posteriormente, seguiu-se a leitura dos títulos e resumos, tendo sido excluídos 1.110 artigos. Foram lidos na íntegra 18 artigos, sendo que 10 foram rejeitados por não se enquadrarem nos critérios de inclusão e 1 pelo desenho do estudo. A amostra final constituiu-se de 7 artigos. Os resultados da pesquisa são apresentados sob a forma de fluxograma PRISMA^(17)^ (Figura 1). As bases onde não se obtiveram resultados não foram retratadas.

**Figura 1:** Fluxograma de identificação, seleção e inclusão dos estudos da metassíntese (PRISMA).

Os dados foram extraídos por dois revisores, utilizando a ferramenta de evidências qualitativas para extração de dados, contendo detalhes da população, contexto, localização geográfica, método de estudo e fenômeno de interesse. A extração dos dados ocorreu de dois modos, em nível do tema e subtema, contidos nos artigos selecionados e cada descoberta foi extraída acompanhada de uma ilustração, ou seja, de uma fala dos sujeitos que participaram das referidas pesquisas. A equipe de revisão (CA e AS) analisou cada descoberta extraída e agrupou-as em categorias, com base na semelhança de sentido de cada uma delas. Para facilitar a visualização, foi inicialmente descrito em folhas de papel e cada revisor (CA e AS) analisou as semelhanças de sentidos de forma separada; em seguida, foi realizado um trabalho de comparação em conjunto para o alcance do consenso^(18)^. As discordâncias foram resolvidas por discussão e consenso com outro revisor (SM).

Os estudos elegíveis foram avaliados criticamente por dois revisores (CA e AS) independentes quanto à qualidade metodológica, utilizando a lista de verificação de avaliação crítica padrão do JBI^(15)^ para investigação qualitativa, Critical Appraisal Checklist for Interpretive & Critical research (QARI). A equipe de investigadores estabeleceu-se como ponte de corte 70%. Os 7 artigos têm respostas positivas entre 80% e 90% no QARI (Quadro 1). Os resultados de cada um dos estudos incluídos em sua avaliação crítica remetem para as questões relacionadas à congruência entre metodologia, objetivos, métodos e evidência de aprovação ética. Observamos algumas limitações relativas à representação e análise dos resultados, localização teórica e cultural do pesquisador e da sua influência na pesquisa.

**Quadro 1:** Avaliação crítica dos estudos elegíveis – Santarém, Portugal, 2023.

Autores	Q1	Q2	Q3	Q4	Q5	Q6	Q7	Q8	Q9	Q10	QARI
Enyert e Burman, 1999^(19)^	Y	Y	Y	Y	Y	U	Y	Y	Y	Y	90%
Donovan et al, 2011^(20)^	Y	Y	Y	U	Y	Y	U	Y	Y	Y	80%
Pope, 2013^(21)^	Y	Y	Y	Y	U	Y	Y	Y	Y	Y	90%
Johansson et al, 2014^(22)^	Y	Y	Y	Y	Y	U	Y	Y	Y	Y	90%
MiIigan e Morbey, 2016^(23)^	Y	Y	Y	Y	U	Y	Y	Y	Y	Y	90%
Saunders e Groh, 2020^(24)^	Y	Y	Y	Y	U	Y	Y	Y	Y	Y	90%
Tay et al, 2021^(25)^	Y	Y	Y	U	Y	Y	U	Y	Y	Y	80%
	100%	100%	100%	80%	70%	80%	80%	100%	100%	100%	

Y = Yes/Sim, U = Unclear/Não está claro, N = No/não.

Q1. Há congruência entre a perspectiva filosófica declarada e a metodologia de pesquisa?; Q2. Existe congruência entre a metodologia de pesquisa e a questão ou objetivos de pesquisa?; Q3. Existe congruência entre a metodologia de pesquisa e os métodos usados para coletar os dados?; Q4. Há congruência entre a metodologia da pesquisa e a representação e análise dos dados?; Q5. Há congruência entre a metodologia da pesquisa e a interpretação dos resultados?; Q6. Existe uma declaração localizando o pesquisador culturalmente ou teoricamente?; Q7. A influência do pesquisador na pesquisa e vice-versa é abordada?; Q8. Os participantes e suas vozes estão adequadamente representados?; Q9. A pesquisa é ética de acordo com os critérios atuais ou, para estudos recentes, há evidência de aprovação ética por órgão apropriado?; Q10. As conclusões tiradas no relatório de pesquisa decorrem da análise ou interpretação dos dados?

### Análise e Tratamento de Dados

Na extração de dados foi estabelecido um nível de credibilidade para cada descoberta, e os resultados foram sintetizados, representando a agregação dos dados dos estudos primários^(18)^.

Os resultados da investigação qualitativa foram agrupados de acordo com a abordagem de meta-agregação do JBI^(14,15)^. Neste processo de agregação, conforme estabelece o JBI^(14,15)^, os achados, por semelhança descritiva e conceitual, foram agregados em subtemas, os quais, posteriormente, foram agregados em temas mais amplos e abrangentes. Na procura de semelhanças e diferenças entre as diferentes perspectivas dos participantes que vivenciaram o fenômeno em estudo, foram integrados os subtemas e os temas que conduziram ao encontro dos metatemas^(18)^. Todas as descobertas foram classificadas de acordo com os níveis de credibilidade do JBI como “inequívocas” (I), “credíveis” (C) ou “sem suporte” (SS). Ambos os revisores (CA e AS) avaliaram os achados extraídos e chegaram a um acordo sobre seus níveis atribuídos de credibilidade. Foram avaliados como “inequívocas” as descobertas acompanhadas por uma ilustração que está além de qualquer dúvida razoável e, portanto, não está aberta a contestação. Os achados avaliados como “credíveis” corresponderam àqueles cujas descobertas estão acompanhadas por uma ilustração sem associação clara e, portanto, aberta ao desafio. Os achados “não suportados” foram aqueles cujas descobertas não são suportadas pelos dados. Apenas achados classificados como “inequívocos” ou “credíveis” foram incluídos na agregação, conforme abordagem do JBI^(15)^. A partir das classificações dos achados e ilustrações, os revisores criaram categorias que, unidas, produziram um único conjunto abrangente de descobertas (Quadro 2).

**Quadro 2:** Processo de agregação dos dados primários – Santarém, Portugal, 2023.

Achados	Subtemas	Temas	Metatema
“Não, não foi difícil, como eu disse, senti que era uma honra cuidar dela, fazer o que eu podia fazer”^(19)^.	Perspectiva de cuidar (I)	Atribuição de sentido (C)	**Necessidades de apoio no desenvolvimento pessoal**
“Estava demasiado ocupado a fazer outras coisas. E agora, na vida real, as coisas importam mais”^(19)^.	Visão da vida (C)		
“Saber que cuidei dele da melhor maneira que pude”^(24)^.	Sem ressentimentos (C)		
“Estou a meditar todas as noites antes de me deitar”^(24)^.	Tempo para mim (C)	Perspectivas de Autocuidado (C)	
“Se não está a fazer exercício, tem de encontrar algo que goste de fazer fisicamente. Encontre uma forma de aliviar o seu stress, seja criativo e mantenha pessoas saudáveis e positivas à sua volta“^(21)^.	Procurar condições de vida (I)		
“Manter a minha versão de comunidade e, com sorte, entrar numa situação em que haverá muitas pessoas por perto, incluindo outras da minha idade“^(21)^.			
“Muitas vezes descubro que eles não se identificam como cuidadores”^(23)^.	Explorar a identidade dos cuidadores masculinos (C)	Reconstrução identitária (C)	
“O termo aconselhamento tem um certo estigma e eu teria receio de revelar o facto de estar a receber um serviço deste tipo aos meus amigos homens e colegas de trabalho”^(23)^.	Masculinidade, emoção e cuidado (C)		
“Dava-me jeito que alguém viesse durante mais umas semanas para manter as coisas normalizadas”^(24)^.	Continuar a ser apoiado (I)	Descontinuidade do apoio na trajetória do cuidar (I)	**Necessidades de apoio na gestão do legado de cuidar**
“Quer seja um pastor ou uma enfermeira, apenas alguém com quem falar - isso teria sido útil”^(24)^.			
“Sinto-me isolada e o hospício cancelou a sessão de aconselhamento para a qual me tinha inscrito”^(25)^.	Solidão e luto no Isolamento (I)		
“Isso (a prestação de cuidados) tem-me afetado muito financeiramente. Estou a viver com menos de metade. Deixei de comprar revistas e outras coisas que costumávamos ter. O meu maior desafio é financeiro”^(24)^.	Fracos recursos financeiros (I)	vulnerabilidade financeira (I)	
“Durante os momentos de trégua, os pensamentos ainda estavam preocupados com as preocupações com o marido”^(24)^.	Persistência da preocupação no cuidar (C)	Impacto na saúde mental (I)	
“Os amigos deixaram de cá vir. Acho que essa foi a parte mais difícil, não ter apoio da família. Afastei-me de tudo porque não podia cuidar dele e manter os meus círculos sociais”^(24)^.	Ausência de relações sociais (I)		
“Estou sozinha, tenho saudades dele. Não ter alguém para cuidar ou mimar, isso é um aspecto negativo”^(24)^.	Tristezas da Solidão (I)		
“Depois fiquei mesmo em baixo durante algum tempo”^(20)^.	Experiência da perda (C)		
Ao contrário dos meus pais, em termos financeiros, espero que o que possamos fazer seja ir para um sítio onde, eventualmente, nos possamos sustentar“^(21)^.	Plano para a vida futura (I)	Projeção de futuro (I)	**Necessidades de respostas formais e informais para o futuro**
“Quero voltar a relacionar-me com as pessoas numa base contínua”^(24)^.	Construir uma rede (I)		
“Foram os grupos de apoio que me ajudaram”^(24)^.			
“Tenho de vos dizer que lidar com a burocracia é muito, muito frustrante e isso é, sabem, para além da dor que estou a sentir, não é divertido. Gostava que alguém me tivesse avisado do que aí vem”^(25)^.	Seguir em frente perante a incerteza Gerir o desconhecido (I)	Serviços estruturados (I)	
“A única altura em que se obtém apoio é quando te queixas, após a morte de alguém, e devia-se tê-lo antes disso, para se preparar para isso”^(19)^.	Disponibilidade da rede de suporte (I)		
“Vou ao grupo de apoio de 15 em 15 dias. É útil para mim conhecer e partilhar experiências com outras pessoas. Estamos, por assim dizer, em diferentes fases da nossa situação de prestação de cuidados, com os nossos cônjuges em centros de dia, em centros de cuidados temporários e em lares de idosos”^(22)^.	Rede social de apoio (I)	Apoio Familiar e comunitário (I)	
“Sento-me aqui sozinho à mesa da cozinha e sinto-me extremamente só. Visitar amigos próximos é muito importante”^(22)^.			
“A minha fé impede-me de tirar a minha própria vida”^(24)^.	Crenças religiosas e estratégias de sobrevivência (C)		
A igreja está a ajudar-me, estou a ir bem mês a mês, a não ser que surja alguma coisa”^(20)^.	Suporte Espiritual (I)		
“Se compreender os imigrantes holandeses, eles vieram em família e, basicamente, durante todo o seu tempo era passado na igreja, também a sua família são os seus amigos. Eles não se relacionam com mais ninguém”^(20)^.	Comportamentos de procura de cuidados culturalmente informados (I)		

(I) “inequívocas”, (C) “credíveis” ou (SS) “sem suporte”.

O processo de agrupamento das categorias gera uma meta-agregação que é apresentada em formato de síntese descritiva, conforme se apresenta no Quadro 2. Os resultados foram utilizados para dar suporte ao Quadro1, com abordagem ConQual^(15)^, e à etapa de discussão, que inclui recomendações de acordo com o GRADE^(15)^, as implicações para a prática e as limitações da revisão.

Na avaliação da confiança das descobertas qualitativas sintetizadas, os achados sintetizados foram classificados de acordo com a abordagem ConQual^(15)^. Cada artigo é inicialmente classificado de alto, moderado, baixo e muito baixo – artigos qualitativos são classificados como alto inicialmente. A partir desse ponto de partida, os mesmos são avaliados nos requisitos confiabilidade e credibilidade^(15)^. A confiabilidade é baseada nas primeiras cinco perguntas do instrumento de avaliação crítica para estudos qualitativos. Essas questões estão relacionadas à adequação da condução da pesquisa com seus objetivos e propósitos. Conforme o número de respostas “sim” para as questões de 1 a 5, a classificação por artigo sobe ou desce (ou permanece a mesma): i) 4–5 respostas “sim”, a avaliação permanece inalterada; ii) 2-3 respostas “sim”, a avaliação desce 1 nível; iii) 0-1 respostas “sim”, a avaliação desce 2 níveis^(15)^. Para a pontuação de credibilidade, os revisores identificaram o nível atribuído para as descobertas que foram sintetizadas, verificando quantas descobertas e qual nível foi atribuído. Para cada descoberta, os revisores atribuíram um dos níveis: Inequívoco (I); Credível (C); Sem suporte (SS). O processo de classificação considera o seguinte cálculo de pontuação para cada descoberta sintetizada: i) Todos os achados inequívocos: o grau permanece inalterado; ii) Combinação de achados inequívocos/credíveis: um nível é rebaixado (-1); iii) Descobertas credíveis/não suportadas: rebaixado três (-3)^(15)^.

## RESULTADOS

Os estudos incluídos na amostra foram realizados entre os anos de 1999 e 2021, sendo um no Canadá, um na Suécia, um no Reino Unido e quatro nos Estados Unidos (Quadro 3). A amostra total de pós-cuidadores foi de 82 (Quadro 3).

**Quadro 3:** Quadro-síntese das características dos estudos incluídos na metassíntese de acordo com os autores, ano de publicação, país, participantes, contexto, fenômeno de interesse e método de análise – Santarém, Portugal, 2023.

Autor/Ano	País	Participantes	Contexto	Fenômeno de interesse	Método de análise
Enyert e Burman, 1999^(19)^	Estados Unidos	7 pós-cuidadores informais; idade 49-68;	Cuidados paliativos	Capacidade de transcender e encontrar significado na experiência de cuidar	Grounded Theory
Donovan et al, 2011^(20)^	Canada	7 , pós-cuidadores informais (cristãos reformados); idade 46-62;	Cuidados Paliativos	Experiência de cuidar e de luto num contexto cultural	Análise de conteúdo
Pope, 2013^(21)^	Estados Unidos	15 mulheres pós-cuidadoras; idade 50-65	Comunitário, agência local de apoio no envelhecimento	Experiência de cuidar e de planejar o seu próprio envelhecimento	Grounded Theory
Johansson et al, 2014^(22)^	Suécia	10 pós-cuidadores informais; idade 56-86	Comunitário, institucionalização da pessoa cuidada	Experiência de cuidar nas decisões de transferência de cuidado	Análise de conteúdo
MiIigan e Morbey, 2016^(23)^	Reino Unido	15 homens pós-cuidadores informais; idade 56-86	Centros Comunitários de cuidadores informais	Experiência do impacto de cuidar na rede de suporte e na identidade	Análise Temática
Saunders e Groh, 2020^(24)^	Estados Unidos	22 viúvas pós cuidadoras; idade média 80 anos	Comunitário, grupo de viúvas de contexto rural e urbano	Experiência do impacto de cuidar na rede de suporte e na viuvez	Análise temática
Tay et al, 2021^(25)^	Estados Unidos	6 pós-cuidadores informais; idade média 32-67;	Comunitário, pós COVID19	Experiência do impacto de cuidar numa época de distanciamento social	Análise de conteúdo

De acordo com o objetivo estabelecido, a revisão sistemática permitiu a descrição subjetiva das experiências e perspectivas do pós-cuidador informal sobre a rede de suporte após o cuidado. A revisão incluiu a produção científica de 24 anos, identificou e analisou 7 estudos qualitativos. Foram extraídos nove (9) achados qualitativos, sendo que seis (6) dos achados foram avaliados como inequívocos e três (3) como credíveis.

O conjunto de achados foi agrupado numa categoria e três subcategorias construídas com base nas divergências, similaridades ou complementariedades. A Figura 2 apresenta o agrupamento de categorias que gerou a seguinte síntese: a necessidade de apoio do pós-cuidador informal.

**Figura 2:** Meta-agregação e resultado da metassíntese em que I – Inequívocas e C – Credíveis.

O Quadro 4 evidencia a síntese dos achados com o Score ConQual. A síntese dos achados apresenta alta confiabilidade, uma vez que o resultado da avaliação da qualidade dos estudos apresentou resposta sim para mais de 4 critérios. Essa classificação forneceu um grau de confiança “moderado” para a descoberta sintetizada, devido a problemas de confiabilidade (a maioria dos estudos não tinha nenhuma declaração de localização do pesquisador e nenhum reconhecimento da influência do autor na pesquisa). No entanto, a credibilidade foi avaliada como moderada, uma vez que as ilustrações foram classificadas como inequívocas e credíveis, ou seja, nem todas representaram significativamente o achado apresentado nos estudos primários.

**Quadro 4:** ConQual Síntese dos achados – Santarém, Portugal, 2023.

Título: Experiências e perspectivas do pós-cuidador informal da rede de suporte				
Participantes: pós-cuidadores informais Fenômeno de interesse: experiências e perspectivas da rede de suporte Contexto: estruturas, grupo e/ou contextos na comunidade				
**Síntese dos achados**	**Tipo de pesquisa**	**Confiabilidade**	**Credibilidade**	**Score ConQual**
Necessidade de apoio ao pós-cuidador informal	Qualitativo	Alta (4–5 respostas “sim”)	Moderada (a combinação de achados inequívocos/credíveis rebaixou um grau (–1))	Moderado 6 (U) + 3 (C)

A análise dos estudos resultou na categoria de necessidade de apoio ao pós-cuidador com três subcategorias que embasaram a construção da meta-agregação, que serão em seguida apresentadas: 1) Necessidade de apoio no desenvolvimento pessoal; 2) Necessidade de apoio na gestão do legado de cuidar; 3) Necessidade de recursos formais e informais para o futuro (Figura 3).

**Figura 3:** Relação dos temas identificados.

### Necessidade de Apoio no Desenvolvimento Pessoal

Este tema agrega as percepções de necessidade de apoio no desenvolvimento pessoal. De acordo com os estudos incluídos, as necessidades traduzem-se em: atribuição de sentido, perspectivas de autocuidado e reconstrução identitária.

A atribuição de sentido para os pós-cuidadores informais caracteriza-se pela necessidade de apoio no desenvolvimento pessoal pela perspectiva de cuidar, visão da vida e sem/ausência de ressentimentos^(19,24)^.

A necessidade de atribuição de sentido abrange a busca de significado para o que foi vivido. Nos diferentes estudos^(19,24)^ está presente a ideia de que houve uma pausa na vida para se tornar cuidador, e depois, quando deixa de cuidar, a necessidade de buscar sentido para aquilo que foi vivido, como essencial. A atribuição de sentido na visão da vida foi reforçada ou reconfirmada pela experiência de cuidar. A visão de vida foi utilizada como estratégia para manter ou restaurar o equilíbrio, resultando num sentido de coerência, durante e após a sua experiência de cuidar, que se pretende sem ressentimentos^(19,24)^. Este processo coloca em perspectiva o que foi vivido, reconhecendo-lhe sentido, o que favorece a ausência de ressentimentos.

Na necessidade de apoio para o desenvolvimento pessoal está ainda presente a perspectiva de autocuidado que se caracteriza por: tempo para mim (si próprio) e procura de condições de vida^(21,24)^.

Para o pós-cuidador o tempo para mim reverte-se no foco dele próprio, na dedicação do tempo para si, para a realização de atividades voltadas ao seu próprio bem-estar, tais como meditação^(21,24)^. A procura de condições de vida está associada às perspectivas de autocuidado, na medida em que expressa o movimento que o pós-cuidador mantém com outras pessoas/outras atividades^(21,24)^.

A reconstrução identitária para os pós-cuidadores informais caracteriza-se pela necessidade de apoio no desenvolvimento pessoal e tem expressão na identidade dos cuidadores, na masculinidade, emoção e cuidado^(23)^. A análise realizada permitiu identificar a identidade dos cuidadores como essencial para a reconstrução identitária e, consequentemente, para o seu desenvolvimento pessoal^(23)^. Acrescentando-se a masculinidade vs feminilidade, emoção e cuidado, aspetos inclusos na reconstrução identitária^(23)^.

### Necessidade de Apoio na Gestão do Legado de Cuidar

A necessidade de apoio na gestão do legado de cuidar remete para marcas, memórias e mudanças associadas ao papel de cuidador informal que persistem. Neste metatema agregam-se as percepções da necessidade de apoio na gestão do legado de cuidar, que se traduzem em: descontinuidade do apoio na trajetória do cuidar, vulnerabilidade financeira e impacto na saúde mental.

A descontinuidade do apoio na trajetória dos cuidadores para os pós-cuidadores permanece mesmo após a perda. A valorização de se manterem apoiados é uma necessidade expressa no discurso escrito nos estudos em análise, principalmente em mulheres e na sua transição para a viuvez^(24)^.

A solidão na perspectiva do isolamento no luto associa-se à ausência de cuidar, apontadas como fatores dificultadores no restabelecimento do sentido da vida: falta de intimidade, adaptação a estar sozinha, ou deixar de ser cuidadora^(24)^.

A vulnerabilidade financeira e/ou fracos recursos financeiros destacam-se como sendo um dos maiores desafios apontados pelos pós-cuidadores, influenciando as suas opções de vida; contudo, na maioria das situações, havia ajuda nas diferentes comunidades por meios dos grupos/redes de apoio^(24)^.

O impacto na saúde mental decorrente da experiência de cuidador informal tem relevância no seu sentido de vida pós-cuidador, ocorrendo a transição de acordo com a história de vida de cada pessoa/família/comunidade. Os estudos analisados mostram que o sentimento da preocupação do cuidar está presente mesmo após a perda, a ausência de relações sociais, a tristeza e a solidão, sentimentos esses que permanecem durante algum tempo. O impacto na saúde mental dos pós-cuidadores parece ter influência na transição do cuidado para a viuvez em dois grandes temas: “time for me now” e “Loneliness”^(24)^.

### Necessidades de Respostas formais e informais para o futuro

Este tema agrega as percepções de necessidade de respostas formais e informais para o futuro. De acordo com os estudos incluídos, as necessidades traduzem-se em: projeção de futuro, serviços estruturados e apoio familiar e comunitário. A projeção de futuro para os pós-cuidadores informais está implícita na necessidade de respostas formais e informais para o futuro e caracteriza-se pela necessidade de criação de um projeto/plano de vida e de construção de uma rede de apoio^(19,21)^.

A projeção de futuro abrange a visão da vida que segue no plano social, comunitário e financeiro^(19,21)^. Nos estudos^(19,21)^, está presente a ideia de que o papel de cuidador informal vulnerabiliza a situação social, como a perda de relações de proximidade – as relações comunitárias manifestadas pelo isolamento de relações e atividades comunitárias – a situação financeira, pela perda de capital, baixos recursos e dificuldade em retomar o mercado de trabalho. A construção da rede aparece como estratégia de projeção de futuro, estando presente a vontade de retomar o relacionamento interpessoal, rompendo com o isolamento e valorizando a participação em grupos de apoio^(19,21)^.

A ausência de serviços estruturados demonstra a necessidade de apoio para as respostas formais e informais para o futuro, para a gestão do desconhecido e para a disponibilidade da rede de suporte^(19,25)^. Gerir o desconhecido representa a ausência de conhecimento do que pode ocorrer após o falecimento da pessoa alvo de cuidados. Esta necessidade relaciona-se com a disponibilidade da rede de suporte existente que, de acordo com os estudos analisados, não está alinhada com as demandas do pós-cuidador^(25)^. O apoio familiar e comunitário para os pós-cuidadores informais inclui a rede social de apoio, crenças religiosas, suporte espiritual e culturas familiares^(20,22,24)^. Nos estudos analisados, a rede social de apoio é essencial na retomada das relações interpessoais e na partilha de experiências. As crenças religiosas são identificadas como âncora na gestão do sofrimento e como ponte para a relação com estrutura religiosa. Por fim, as culturas familiares revelam-se basilares no sentimento de segurança e apoio, no decorrer da trajetória de cuidar^(20,22,24)^.

## DISCUSSÃO

Esta revisão sistemática com metassíntese foi realizada para melhor compreender as experiências e perspectivas do pós-cuidador informal sobre a rede de suporte após o cuidado. Sete (7) estudos foram incluídos após um rigoroso processo de busca e seleção. Todos os estudos incluídos são de elevada qualidade, com base na lista de verificação de avaliação crítica do JBI para investigação qualitativa (Quadro 1).

A produção científica incluída na revisão decorre no período de 1999 a 2021, e os estudos incluídos envolvem investigadores dos EUA, Canadá, Suécia e Reino Unido (Quadro 3). A evidência da qualidade é moderada (Quadro 4).

Destaca-se que nenhum estudo foi realizado em Portugal, o que é relevante, pois a localização dos estudos pode enfatizar a preocupação com os pós-cuidadores informais. Assim, foi possível identificar que o interesse por esta temática envolve pesquisadores de países de vários continentes, com diferentes olhares sobre o fenômeno, considerando os padrões culturais, econômicos e sociais na sua compreensão. Por outro lado, a realidade dos países em análise ressalta que a temática em estudo seja uma preocupação ainda muito focalizada nos países onde se valoriza o interesse no agravamento do envelhecimento e no cuidado informal. O contexto dos estudos é comunitário, em cuidados paliativos e em contexto de institucionalização, relevando a importância da família, amigos e estruturas comunitárias como a igreja^(19,20,21,22,23,24,25)^.

A metassíntese identificou e forneceu uma compreensão das experiências e perspectivas do pós-cuidador informal sobre a rede de suporte após o cuidado e comparou-as com a literatura disponível. A percepção dos pós-cuidadores relativamente à rede de suporte é de ausência de identificação das suas necessidades individuais e interpessoais, bem como de desarticulação dos serviços formais e informais^(19,20,21,22,23,24,25)^.

Os estudos incluídos na revisão chamam a atenção para a necessidade de apoio dos pós-cuidadores no desenvolvimento pessoal. Nestes estudos é explorada a experiência de cuidar e o modo como altera a forma de perspectivar a vida, apontando para a importância da valorização do apoio na reconstrução identitária e na integração da experiência de cuidar na vida após o cuidado. Este achado corrobora os resultados do estudo de Lennaerts-Kats et al.^(26)^ que tinha o objetivo de compreender as experiências dos cuidadores familiares enlutados durante o período de cuidados informais na fase de cuidados paliativos, bem como após a morte do seu ente. No mesmo estudo foi identificado que os pós-cuidadores sentem alteração da sua saúde mental durante muitos anos antes e depois da morte da pessoa cuidada. A vulnerabilidade do pós-cuidador informal, identificada na revisão, vai ao encontro do que é retratado em estudos prévios^(3,4,5,6,7)^. Os pós-cuidadores informais aparecem identificados como enlutados e/ou ex-cuidadores, com prevalência no gênero feminino^(19,20,21,23,24)^, embora um estudo tenha o seu foco em pós-cuidadores masculinos^(25)^. Identificou-se que o processo de prestação de cuidados e o seu impacto na vida após a cessação de cuidados é manifesto na esfera pessoal, com perturbações na vida e nas relações. Reconheceu-se que este processo potencializa a alteração dos seus papéis sociais e relacionais, podendo levar ao afastamento da identidade, cristalizada no papel de cuidador, bem como com a alteração da saúde pessoal^(19,24)^. Na esfera interpessoal é clara a perda de relações sociais e a vontade de retomada da atividade social, embora a realidade atual seja diferente da realidade quando da assunção do papel de cuidador^(19,20,21,22,23,24,25)^. Esta análise evidencia a importância de atender e valorizar a experiência no percurso de cuidador.

Relativamente aos achados da necessidade de apoio no impacto do legado de cuidar, como na saúde do cuidador, referem-se aos pós-cuidadores informais pelas taxas mais altas de estresse^(27)^, depressão^(28)^ e problemas de sono^(29–30)^. Do mesmo modo, o cuidado informal perturba o sono de várias formas^(30,31)^, sendo que as perturbações do sono persistem após a cessação de cuidados. Esta fragilidade da saúde física e mental do cuidador permanece no pós-cuidador com implicações no processo de luto^(8)^. Estes aspectos reforçam a importância da permanência de contato com profissionais de saúde após a cessação de cuidados.

A necessidade de apoio nos recursos formais e informais é identificada nos estudos incluídos na revisão, por meio da qualidade de vida, ganhos em saúde, contato com a comunidade e família, reintegração no mercado de trabalho, suporte econômico, ocupação, bem-estar emocional, ligação com os serviços de saúde e apoio a outros cuidadores. Os artigos fazem uma abordagem à pessoa na sua globalidade^(7)^, dando evidência ao percurso de cuidar^(19,20,21,22,23,24,25)^ do pós-cuidador. Esta necessidade é identificada na comunidade, relevando a importância da família, amigos e estruturas comunitárias como a igreja^(19,20,21,22,23,24,25)^. Estas referências reforçam o enquadramento do apoio ao pós-cuidador familiar em contexto comunitário.

No percurso da revisão sistemática com metassíntese desenvolvida, verificou-se a relevância das evidências de estudos qualitativos sobre as experiências e perspectivas dos pós-cuidadores informais da rede de suporte: necessidade de apoio no desenvolvimento pessoal; necessidade de apoio na gestão do impacto do legado de cuidar; necessidade de recursos formais e informais. Estes achados confirmam a necessidade de acolhimento e qualificação dessas experiências na estruturação de uma rede de suporte. A metassíntese revelou lacunas relacionadas com a necessidade de apoio, como no desenvolvimento pessoal, na gestão do impacto do legado de cuidar e nos recursos formais e informais. Deste modo, com este estudo, a proposta estratégica de desenvolvimento da rede de suporte deve assentar nas necessidades de apoio identificadas.

No que diz respeito às implicações para a prática, os achados sugerem que intervenções focadas em melhorar as necessidades de apoio dos pós-cuidadores podem ser benéficas na estruturação de uma rede de apoio. Os pilares da rede devem assentar nas necessidades de apoio do desenvolvimento pessoal, na gestão do impacto do legado de cuidar e nos recursos formais e informais para o futuro. O papel dos profissionais de saúde, especialmente os enfermeiros especialistas, é essencial para encorajar a vigilância da saúde, com foco na reflexão e discussões sobre a experiência, o significado percebido e ainda, o identificar potenciais sistemas de apoio e disponibilidade dos pós-cuidadores. Esta vigilância poder-se-ia perspectivar numa consulta estruturada por profissionais treinados para as especificidades do pós-cuidador-informal. Acrescenta-se a importância da integração da rede de suporte numa perspectiva holística^(13)^ do pós-cuidador, procurando respostas contínuas na comunidade^(3,7)^.

Contudo, as recomendações são feitas com prudência devido à qualidade moderada da evidência (Quadro 4). De acordo com os resultados obtidos, seguindo o GRADE do JBI^(14)^, recomenda-se que (1) a rede de suporte ao pós-cuidador informal seja em contexto comunitário^(19,20,21,22,23,24,25)^; (2) as necessidades de apoio no desenvolvimento pessoal, na gestão do impacto do legado de cuidar e nos recursos formais e informais sejam colmatadas^(19,20,21,22,23,24,25)^; (3) as percepções e experiências enquanto cuidadores sejam valorizadas^(19,20,21,22,23,24,25)^.

Algumas limitações do estudo são identificadas: 1) Número reduzido de estudos incluídos. Não tendo a equipe de investigadores colocado limite temporal, denota a reduzida evidência na área. Inclui-se ainda a possibilidade de perda de alguns estudos relevantes em línguas diversas. 2) Os resultados não se destinam a generalizações e devem ser interpretados de acordo com as estruturas, grupo e/ou contextos na comunidade. A realização da revisão sistemática da literatura com metassíntese permitiu, por meio dos vários estudos qualitativos, construir um conhecimento cada vez mais robusto e com maior capacidade de transferência na área do pós-cuidador informal. Contudo, a pesquisa qualitativa fornece asserções teóricas e contextuais sobre as experiências de um número limitado de participantes em ambientes comunitários. 3) Possibilidade de polarização dos resultados, pois a experiência da rede de suporte tem representações discrepantes de acordo com as vivências dos pós-cuidadores e dos contextos comunitários. Contudo, as percepções encontradas nos estudos permitiram expandir o entendimento do fenômeno em investigação, pois os resultados apontaram que, nestes contextos, a diversidade das representações ilustram a necessidade de incluir a rede de suporte na perspectiva holística do pós-cuidador. 4) As limitações descritas intensificam as dificuldades existentes quanto ao fenômeno e à necessidade da realização de novos estudos em diferentes contextos. A generalidade dos estudos incluídos na revisão não abordaram a influência do ambiente e do investigador no desenvolvimento da pesquisa, aspecto que não foi referido.

## CONCLUSÃO

Esta revisão sistemática com metassíntese verificou que as evidências de estudos qualitativos sobre as experiências e as percepções dos pós-cuidadores informais da sua rede de suporte revelam ausência de identificação das suas necessidades individuais e interpessoais, bem como de desarticulação dos serviços formais e informais. Os resultados são consistentes com estudos anteriores dirigidos a cuidadores informais que destacam a importância do suporte social. Os achados desta revisão com metassíntese enfatizam a necessidade de um enfoque na necessidade de apoio aos pós-cuidadores, o que confirma a importância de acolhimento e atenção às necessidades dos pós-cuidadores, particularmente de apoio no desenvolvimento pessoal; de apoio na gestão do impacto do legado de cuidar; e de recursos formais e informais.

Mais estudos são necessárias para explorar a experiência dos pós-cuidadores informais em diferentes contextos culturais. Estudos longitudinais poderiam fornecer uma compreensão mais profunda das mudanças nas experiências ao longo do tempo.

É imperativo que os profissionais de saúde comecem a debater sobre as especificidades das necessidades dos pós-cuidadores e de respostas estruturadas, como a implementação de consultas de vigilância de saúde disponíveis no serviço nacional de saúde. Para isto são necessários profissionais treinados, como enfermeiros especialistas que elevem as competências de acompanhamento específicas de pós-cuidadores informais. Torna-se fundamental, no nível da formação em enfermagem, a introdução nos currículos da temática dos pós cuidadores e da sua rede de suporte na comunidade. Finalmente, sugere-se o desenvolvimento de investigação futura, com o desígnio de: identificar os elementos a mobilizar na construção da rede de suporte; identificar as percepções dos profissionais relativas ao modo de operacionalização da rede; e implementar intervenções que permitam o apoio e acompanhamento do pós-cuidador informal.

Acredita-se que os resultados da revisão com metassíntese terão impacto no desenvolvimento de uma cultura de cuidado enraizada na comunidade, em que as experiências de cuidar sejam valorizadas.

De acordo com os resultados encontrados, parece desejável o desenvolvimento de investigação para a) identificar eixos estratégicos para o desenvolvimento da rede de suporte, replicáveis e adaptáveis aos contextos comunitários; b) implementar intervenções estruturadas com base nas necessidades que permitam a continuidade do acompanhamento do pós-cuidador pela equipe de saúde.
